# 

**Published:** 1890-12-13

**Authors:** 


**The Press and the Hospitals** - 165

**Passing Topics.**—Special Preachers - 166

**The Doctor's Armchair.**—Jenner, Pasteur, and Koch - 167

**The Tomato and Cancer.** By W. T. Fernie, M.D. - 168

**The Consumers' League.** By a Correspondent - 169

**London's Great Problems.**—North Kensington Friendly Workers - 170

**Everybody's Page** - 171

**Notes and News** - 172

**Annotations.**—Dr. Sarah E. Post—An Honorary and an Honourable Secretary—Dr. Rentoul's Crusade Against Midwives - 174

**The Student of Medicine.**—Editorial Notes—Appointments—Students' Medical Societies—Notes from the Schools —Pass Lists—Athletics—Events for December - 180

**The Practitioner's Mirror and Retrospect:—**

The London Post-Graduate Course at the Paddington Infirmary.—Gonorrhœal Arthritis, Gout and Rheumatoid Arthritis - 175

Cambridge University Medical Society - 176

Medicine.—The Relation of Lupus to Tubercle—The Etiology of Empyema - 176

Surgery.—Intubation in Diphtheria—Salt as an Antiseptic —Surgical Treatment of Tuberculous Peritonitis - 177

Therapeutics.—Action of Salol on the Kidneys - 177

The Infirmary Medical Superintendents' Society - 178

Pharmacy.—Lysol - 179

**Books Received** - 179

**Scraps and Gleanings** - 179

**General Charities.**

EXTRA SUPPLEMENT—THE NURSING MIRROR.

**En Passant.**—At Hull—Leprosy Nursing—Nursing in Lunatic Asylums—Short Items—Nurses' Conversazione—A Woman's view - liii

**The London Hospital and its Nurses** - liv

**Amusements and Relaxation** - lviii

**Everybody's Opinion.**—Fever Hospitals—Help Wanted—A Practical Hint—The Trained Nurse in the Nursery - lvii

**Off Duty.**—Domestic Medicine in the 18th Century (*Concluded*) - lviii

**Appointments—Notes and Queries** - lviii

